# Simultaneous retrieval of temperature-dependent absorption coefficient and conductivity of participating media

**DOI:** 10.1038/srep21998

**Published:** 2016-02-25

**Authors:** Yatao Ren, Hong Qi, Fangzhou Zhao, Liming Ruan, Heping Tan

**Affiliations:** 1 School of Energy Science and Engineering, Harbin Institute of Technology, Harbin, P. R. China, 150001

## Abstract

A secondary optimization technique was proposed to estimate the temperature-dependent thermal conductivity and absorption coefficient. In the proposed method, the stochastic particle swarm optimization was applied to solve the inverse problem. The coupled radiation and conduction problem was solved in a 1D absorbing, emitting, but non-scattering slab exposed to a pulse laser. It is found that in the coupled radiation and conduction problem, the temperature response is highly sensitive to conductivity but slightly sensitive to the optical properties. On the contrary, the radiative intensity is highly sensitive to optical properties but slightly sensitive to thermal conductivity. Therefore, the optical and thermal signals should both be considered in the inverse problem to estimate the temperature-dependent properties of the transparent media. On this basis, the temperature-dependent thermal conductivity and absorption coefficient were both estimated accurately by measuring the time-dependent temperature, and radiative response at the boundary of the slab.

The determination of thermal and radiative properties in coupled radiation and conduction problems is highly important for many engineering applications, e.g., thermal protection and insulation system design^1^,^2^, fire-proof materials^3^, photothermal management^4^, and other engineering applications^5^,^6^. In the last decades, the inverse coupled radiation and conduction problems have drawn considerable attention because of their extensive applications^7^,^8^,^9^,^10^. The conduction–radiation parameter, scattering albedo, and/or boundary emissivity were estimated in these studies. In the majority of the studies, the thermal and radiative properties were considered constant. However, for practical application, the thermal and radiative properties, such as thermal conductivity and absorption coefficient, are always considered to be temperature-dependent^11^.

Temperature-dependent optical thermal properties have significant importance in the application of optical materials or gas. For example, in applications like photoluminescence or electroluminescence^12^, the thermal and optical properties of porous silicon play an important role. The thermal and optical properties must be known to optimize the heat generation in these applications^13^. In laser technique, the optical components in high laser intensity always suffer from thermal effect, which affects the sensitivity of the equipment^14^. The noise caused by the temperature change can be predicted accurately if the optical and thermal properties of those components are known. More importantly, the properties of commonly used optical materials, such as silicon, are always temperature-dependent^15^. Hence, this work focuses on the estimation of temperature-dependent thermal conductivity and absorption coefficient of non-scattering media.

The thermal or optical respond signals should be measured first to estimate the properties of the non-scattering media. In recent years, laser technique has been widely applied in various fields, including the inverse radiative transfer problems^16^,^17^. The reason is that the interaction between the laser and the media can generate measurable signals that carry the inner information about the media. For the transient radiation and conduction problems, the laser can lead to temperature rise and radiative responses. Therefore, in the present work, lasers are used to estimate the optical and thermal properties of the media. The time-resolved temperature can be easily and accurately measured with an invasive technique, such as thermocouple^18^, or noninvasive technique, such as infrared thermography^19^. Many techniques for optical signal measurement exist, such as infrared Fourier transform spectrometry^20^ and time-correlated single photon counting technique^21^.

In addition to optical and thermal signal measurement, inversion techniques must be investigated when the properties of the participating systems are estimated. Inversion techniques have been studied thoroughly in the past decades, and they can be roughly classified into two categories^10^: (1) gradient-based techniques, such as the Gauss–Newton^22^,^23^, Levenberg–Marquardt^24^, and conjugate gradient methods^25^; and (2) stochastic heuristic intelligent optimization techniques, such as generic algorithm^9^,^26^, particle swarm optimization (PSO)^27^,^28^, and ant colony optimization^10^,^29^. The stochastic heuristic intelligent optimization techniques are proved powerful techniques for the inverse problems in many areas^10^,^30^. These intelligent algorithms have received increasing attention as new inverse solvers because of their superior aid in stability and in achieving global minimum solutions compared with conventional gradient-based methods, particularly for problems with high dimensions^31^. Our previous work^27^ showed that stochastic PSO (SPSO) performs effectively in the inverse radiative problems to estimate the extinction and scattering coefficients. It was demonstrated to be fast and robust. In this work, SPSO was applied to solve the inverse coupled radiation and conduction problems.

In the present work, temperature-dependent *κ*_a_ and *λ* were retrieved accurately with a secondary optimization method that uses both thermal and optical information. The key point of the proposed method is to have a proper direct model to predict the measurable responds. Then, the temperature-dependent optical and thermal properties can be obtained using the proposed secondary optimization technique by compared the predict data and experiment data. In the present research, a secondary optimization technique is applied to the macroscopic media. But the application of this method can be extended to micro or even nano materials. The remainder of this paper is organized as follows. The transient coupled radiation and conduction model is described in Section 2. The principle of SPSO is introduced in Section 3. The direct model is verified first, and then the inverse problem and sensitivity analysis are presented in Section 4. The main conclusions are provided in Section 5.

## Direct Problem

**Figure 1:**
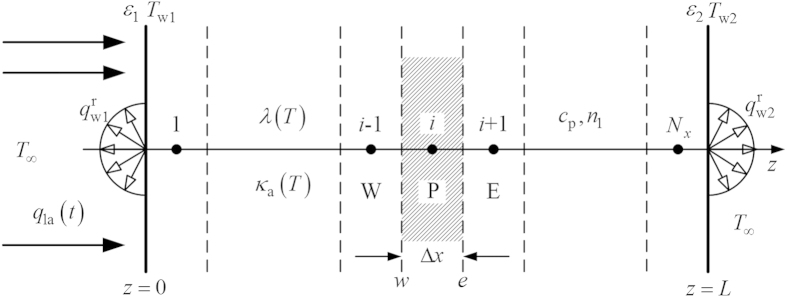
Schematic of the coupled radiation and conduction heat transfer in a slab subjected to a pulse laser.

The transient coupled radiation and conduction heat transfer in an absorbing, emitting, but non-scattering 1D slab are considered (see Fig. 1). The boundaries of the slab are diffuse gray walls. The left surface of the slab is exposed to a pulse laser and the emissivity of the boundaries are 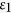 (*z* = 0) and 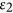 (*z* = *L*), which are set as 1 in this work. The temperature of the environment is constant.

The energy equation governing the transient coupled radiation and conduction heat transfer in a participating medium with temperature-dependent physical parameters is defined as follows^32^:


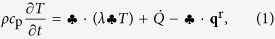


where *T* and *t* denote temperature and time, respectively. The density and specific heat capacity of the media are represented by *ρ* and 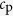, respectively. The thermal conductivity *λ* is considered dependent on temperature *T*. The heat generation inside the media is expressed as 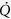, and 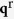 is the radiation heat flux.

The reduced equation for a 1D planar medium without internal heat generation is as follows:


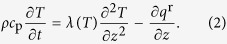


The initial and boundary conditions are as follows:


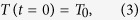



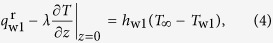



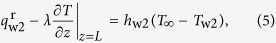


where *T*_w1_ and *T*_w2_ represent the temperature of the left and right walls. The convective heat transfer coefficients in the left and right walls are denoted by *h*_w1_ and *h*_w2_, respectively. *T*_∞_ is the temperature of the environment. 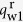 and 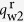 are the radiative intensity in the left and right walls of the media, respectively. The radiative intensity in the boundary and the radiative source term in Eq. (2) can be obtained by solving the radiative transfer equation as follows:


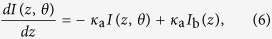


where *I* represents radiation intensity. The subscript b stands for blackbody. *θ* is the polar angle. Then, 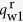, 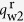, and 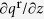 can be expressed as:


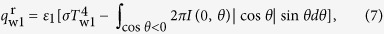



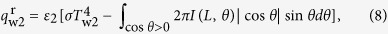



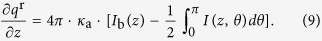


The finite volume method (FVM) was applied to solve the direct coupled radiation and conduction problem. The detailed solution procedure is available in Refs 10 and 32 and will not be repeated here.

## Inverse Problem

PSO algorithm is a population-based optimization algorithm first introduced by Eberhart and Kennedy^33^. The underlying motivation of PSO algorithm development is the social behavior of animals, such as bird flocks and fish schools. The flock population is called a swarm, and the individuals are called particles. One particle indicates a potential solution. The standard PSO has its natural weakness of premature convergence. Many modified PSO algorithms were proposed in recent years to avoid its natural weakness and to ensure overall convergence. PSO-based algorithms are proved adaptive and robust parameter-searching techniques^27^.

In the present work, SPSO was applied to solve the inverse problem. The fundamental idea of SPSO method involves the use of a random generated particle to improve global searching ability. In traditional PSO, each particle adjusts its own position at every iteration to move toward its best position and best neighborhood according to the following equations:


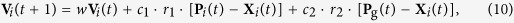



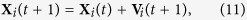


where *c*_1_ and *c*_2_ are two positive constants called acceleration coefficients; *r*_1_ and *r*_2_ are uniformly distributed random numbers in the interval [0, 1]; 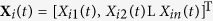 denotes the present location of particle *i*, which represents a potential solution; 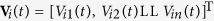 is the present velocity of particle *i*, which is based on its own and its neighbors’ flying experience; 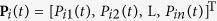 denotes the “local best” in the *t*th generation of the swarm. The global best position of the swarm is expressed as **P**_g_. The inertia weight coefficient *w* is used to regulate the present velocity. This coefficient also influences the tradeoff between the global and local exploration abilities of particles, which means *w* influences the local and global searching ability of the algorithm. Large *w* corresponds to the effective global searching ability and poor local searching ability. If *w* is set as 0, the velocity of the *i*th particle in the next generation is only related to the current position 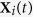 and to the local and global best positions, namely, **P**_*i*_ and **P**_g_, respectively. The following equation can be obtained by substituting Eq. (11) into Eq. (10):


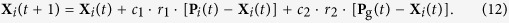


Thus, if 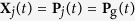, particle *j* stops updating its position, thereby leading to the loss of global searching ability and to the increase of local searching ability. A random generated particle is introduced to improve its global searching ability by replacing particle *j*, whereas other particles update their position according to Eq. (12). Thus, the updating procedure can be described as follows:


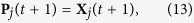



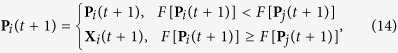



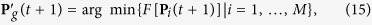



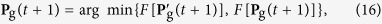


where ***F*** denotes the object function. After the generation of random particle *j*, three different situations occur. (1) If **P**_g_ = **P**_*j*_, the random particle *j* locates at the best position, and the new random particle is generated repeatedly in the searching domain. (2) If **P**_g_ ≠ **P**_*j*_ and the global best position **P**_g_ is not updated, all of the particles are updated with Eq. (12). (3) If **P**_g_ ≠ **P**_*j*_ and the global best position **P**_g_ is updated, a particle *k* (*k* ≠ *j*) with **X**_*k*_ (*t* + 1) = **P**_*k*_ = **P**_g_ must exist; then, particle *k* stops evolving, and a new random particle is generated repeatedly in the searching domain. Thus, at certain generation in the evolutionary procedure, at least one particle *j* satisfying **X**_*j*_ (*t* + 1) = **P**_*j*_ = **P**_g_ exists. This observation indicates that at least one random particle is generated in the searching domain to improve the global searching ability of PSO algorithm^27^.

## Results and Discussion

In this section, the verification of the FVM model used in the present work is presented first. Afterwards, two test cases, which are the retrieval of temperature-independent and temperature-dependent properties, are shown. Each case was implemented with FORTRAN code, and the developed program was executed on an Intel Core i7-3770 PC.

### Verification of FVM model

The results were compared with the work of Tan^34^ to verify the developed FVM code of the transient coupled radiation and conduction problem. A 1D semitransparent gray slab with opaque boundaries exposed a square pulse of 1 s pulse width. The medium was assumed to be non-scattering, and the heat convective was considered in both boundaries. The thermophysical parameter settings are shown in Table 1.

**Figure 2:**
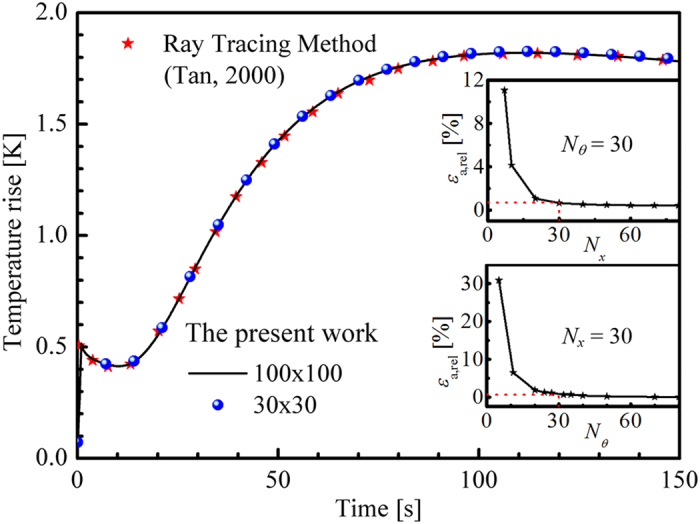
Verification of the direct model. *ε*_a, rel_ is the average relative error of the temperature rise at every time step.

Given that the time step was set as ∆*t* = 0.1 s, no obvious difference was observed in the time-dependent temperature when the numbers of control volume *N*_*x*_ and direction *N*_*θ*_ were both beyond 30, as illustrated in Fig. 2. Therefore, *N*_*x*_ and *N*_*θ*_ were both set as 30 to save computation time without losing accuracy.

### Estimation of absorption coefficient and thermal conductivity

A 1D case of participating media was presented in this work to demonstrate that the absorption coefficient *κ*_a_ and thermal conductivity *λ* could be retrieved from the measured temperature. The direct model is shown in Section 2.1. In this section, the time-dependent temperature in the right side of the 1D slab *T*_w2_ was applied to obtain *κ*_a_ and *λ*. The inverse problem was proceeded by minimizing the objective function, which is expressed as:


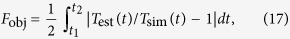


where *T*_est_ is the time-dependent temperature calculated with the estimated properties. The simulated time-dependent temperature is denoted by *T*_sim_. The sampling time interval was [*t*_1_, *t*_2_], which was [0, 150s] in the present work.

The random standard deviation was also added to the signals computed from the direct model to demonstrate the effects of measurement errors on the inversed parameters. The following relation was used in present inverse analysis:


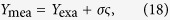


where *Y*_mea_ is the measured value, *Y*_exa_ represents the exact value of the measured signal, and *ζ* is a normal distribution random variable with zero mean and unit standard deviation. The standard deviation of the measured radiative intensities at the boundaries *σ* for a *γ*% measured error at 99% confidence was determined as the follows:


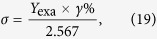


where 2.576 arises because 99% of a normally distributed population is contained within ±2.576 standard deviation of the mean. The relative error 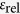 is defined as follows to evaluate the accuracy of the retrieval results:


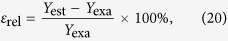


where *Y*_est_ denotes the estimated value calculated with the estimated parameters.

The population size of the SPSO was set as 50. The acceleration coefficients were set as *c*_1_ = *c*_2_ = 1.2. The stopping criteria were set as follows: (1) The iterations reached *t*_max_, which was set as 5000. (2) Or the objective function was smaller than *ε*_0_, which was set as 10^−6^ in the present work. The thickness of the 1D slab was set as 0.01 m, and the pulse width was 5 s. The refractive index was set as 1.8. The absorption coefficient and thermal conductivity of the slab were set as 18.7 m^−1^ and 0.61 W/(m·K), respectively. The searching intervals for the two parameters were set as [0, 50] and [0, 10]. The other parameters were set the same as those presented in Table 1. The retrieval results are shown in Table 2.

Table 2 shows that *κ*_a_ and *λ* could be retrieved accurately even with measurement errors. The results of *λ* were also more accurate than those of *κ*_a_ with or without measurement errors. The reason is that the temperature response was sensitive to *λ*, which indicated that *λ* was important in determining the temperature rise in the right wall of the media. This finding is discussed later in present work.

### Estimation of temperature-dependent absorption coefficient and thermal conductivity

The properties of the media were always temperature-dependent^35^,^36^. Therefore, in this section, we attempted to estimate the temperature-dependent absorption coefficient and thermal conductivity simultaneously. They were set as *κ*_a_(*T*) = *a*_1_ + *a*_2_·*T* = −22.1 + 0.23*T* mm^−1^ and *λ*(*T*) = *b*_1_ + *b*_2_·*T* = 0.012 + 4.09 × 10^−4^*T* W/(m·K), which were the fitting results of porous silicon from refs 13 and 15. The laser intensity was set as 10000 W/m^2^, and the pulse width was set as 5 s. The thickness of the slab was set as 0.005 m. The other parameters of the direct model and inverse model were set the same as those presented in the preceding section. The searching intervals for the four parameters were set as [−50, 50], [0, 1], [0, 0.1], and [0, 0.001]. The retrieval results are shown in Table 3.

The retrieval of the absorption coefficient results was evidently not acceptable. However, the temperature-dependent conductivity could be retrieved accurately. For parameter estimation problems, a detailed examination of the sensitivity coefficients could provide considerable insight into the estimation problem. The sensitivity coefficient was the first derivative of a dependent variable, such as time-resolved transmittance or reflectance, with respect to an independent variable. If sensitivity coefficients were either small or correlated with one another, then the estimation problem was highly sensitive to measurement errors, thereby making the estimated parameters difficult to obtain. The sensitivity coefficient is defined as follows:


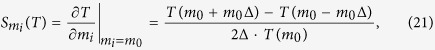


where *m*_*i*_ denotes the independent variable, which stands for *κ*_a_ and *λ* in this study. ∆ represents a tiny change.

**Figure 3:**
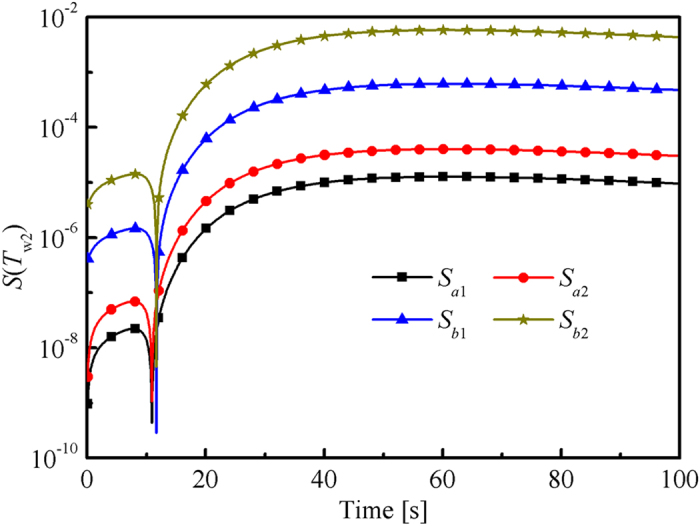
Sensitivity of *T*_w2_ to the parameters that need to be retrieved, in which *κ*_a_(*T*) = *a*_1_ + *a*_2_·*T* = −22.1 + 0.23*T* mm^−1^ and *λ*(*T*) = *b*_1_ + *b*_2_·*T* = 0.012 + 4.09 × 10^−4^*T* W/(m·K).

Figure 3 shows that the sensitivity of *T*_w2_ to *b*_1_ and *b*_2_ was several order of magnitudes larger than that to *a*_1_ and *a*_2_, which indicated that *λ*(*T*) was more important than *κ*_a_(*T*) for the change of temperature response. Therefore, *κ*_a_(*T*) could not adequately have an influence on the value of objective function when the difference of *T*_w2_ was applied. In other words, the stopping criteria (the value of objective function was smaller than *ε*_0_) could be satisfied if the estimated *λ*(*T*) was close to the true value regardless of the estimated *κ*_a_(*T*).

**Figure 4:**
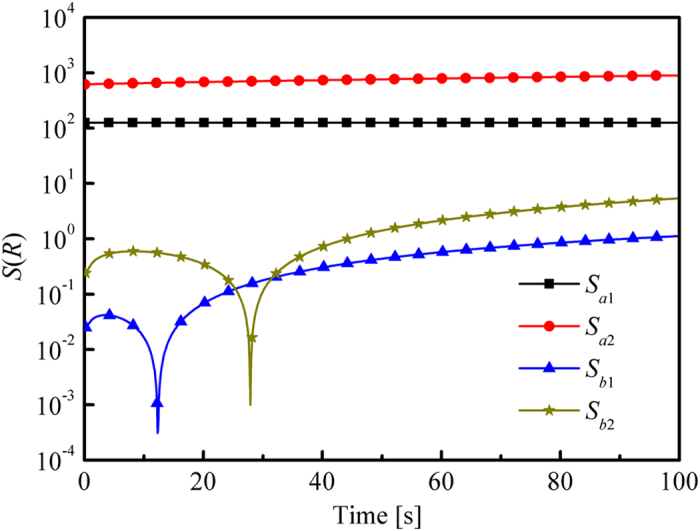
Sensitivity of *R* to the parameters that need to be retrieved, in which *κ*_a_(*T*) = *a*_1_ + *a*_2_·*T* = −22.1 + 0.23*T* mm^−1^ and *λ*(*T*) = *b*_1_ + *b*_2_·*T* = 0.012 + 4.09 × 10^−4^*T* W/(m·K).

Therefore, the accuracy of the estimated *κ*_a_(*T*) must be improved. Thus, other information that was sensitive to *κ*_a_(*T*) should be included in the inverse procedure. The radiative intensity *R* in the right side of the slab was directly related to *κ*_a_(*T*). Thus, the sensitivity of *R* to *κ*_a_(*T*) and *λ*(*T*) was studied (see Fig. 4). Radiative intensity *R* was more sensitive to *κ*_a_(*T*) than to *λ*(*T*). Hence, *R* could be applied to estimate *κ*_a_(*T*).

**Figure 5:**
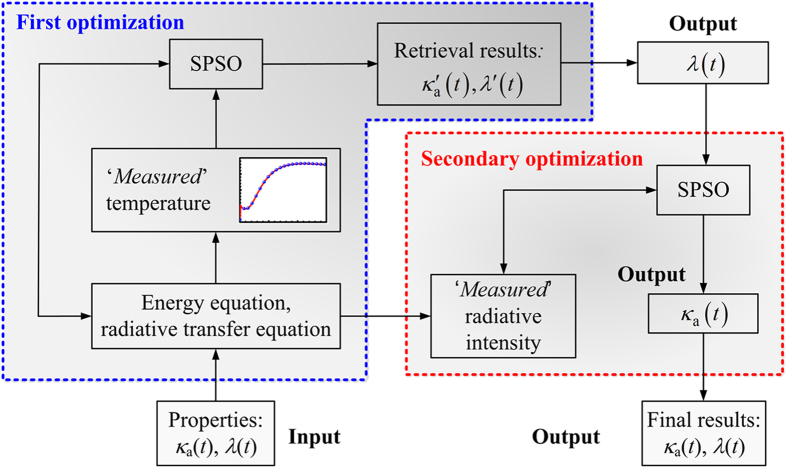
Flowchart of the whole optimization procedure.

**Figure 6:**
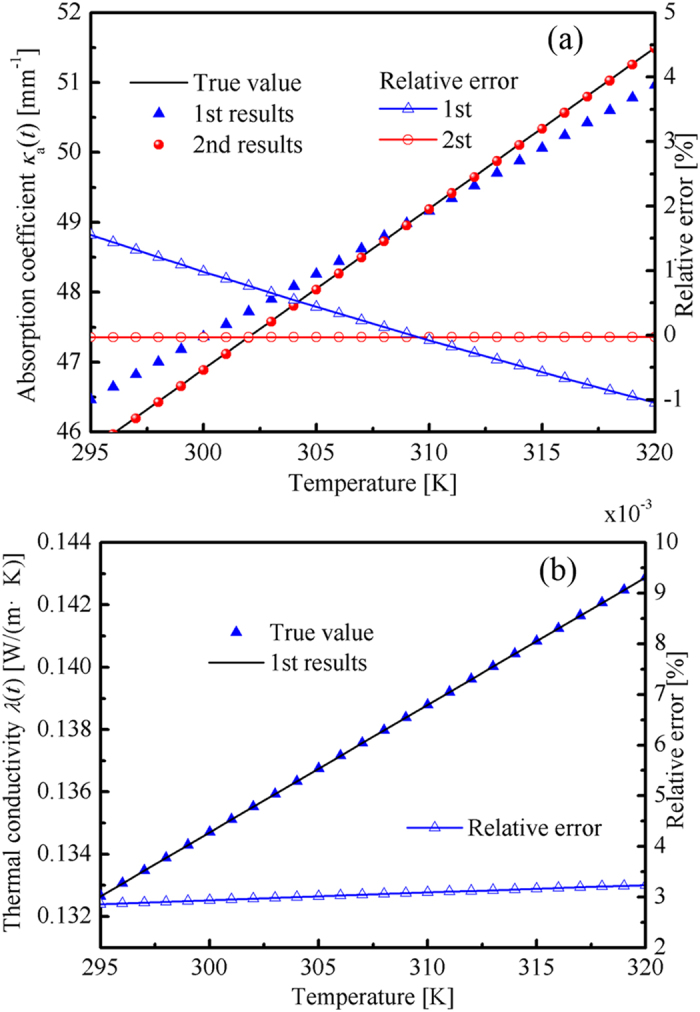
Temperature dependent (**a**) absorption coefficient and (**b**) thermal conductivity.

Based on the preceding analysis, a secondary optimization was performed, in which *a*_1_ and *a*_2_ were parameters that needed to be retrieved again, whereas *b*_1_ and *b*_2_, which were retrieved by the first optimization, were applied as the original values. The flowchart of the whole optimization procedure is shown in Fig. 5 for clarity. The objective function in the secondary optimization is expressed as follows:


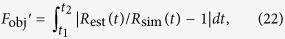


where the simulated and estimated radiative signals are denoted by *R*_sim_ and *R*_est_. In the secondary optimization, the pulse width was set as 100 s. The other parameters were set the same as those mentioned earlier. The results are shown in Table 4 and Fig. 6. The accuracy of the *κ*_a_(*T*) second retrieval results was considerably higher than that of the first retrieval results. The accuracy of the retrieval results was improved significantly. Therefore, the optical and thermal properties could be retrieved accurately when the optical and thermal information were both used in the inverse problem.

**Figure 7:**
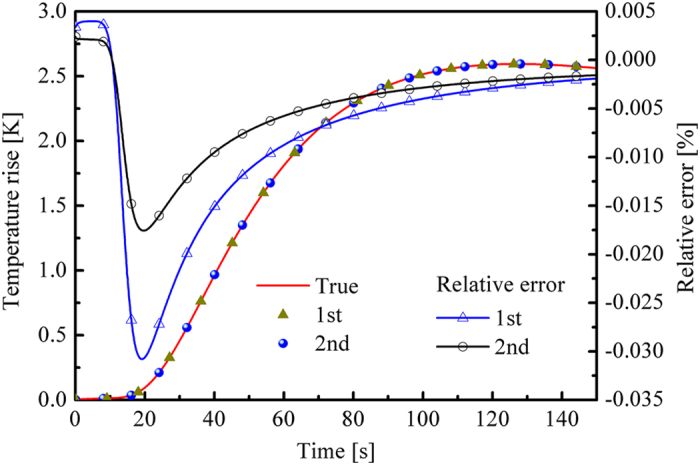
Time-dependent temperature response in the right boundary. The legend “true” means the results are calculated with the real properties (*κ*_a_(*T*) = −22.1 + 0.23·*T* mm^−1^) of the media. “1st” and “2nd” stand for the results calculated with the 1st (*κ*_a_(*T*) = −6.638 + 0.180·*T* mm^−1^) and 2nd (*κ*_a_(*T*) = −22.114 + 0.230·*T* mm^−1^) retrieval results, respectively.

**Figure 8:**
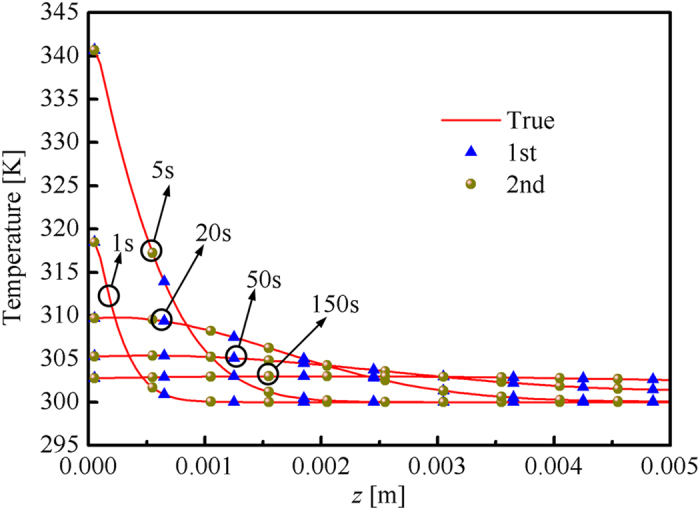
Comparison of temperature distribution in 1, 5, 20, 50, and 150 s.

The conclusion from the sensitive analysis indicated that the stopping criteria (the value of objective function was smaller than *ε*_0_) could be satisfied if the estimated *λ*(*T*) was close to the true value regardless of the value of estimated *κ*_a_(*T*). This conclusion could be verified with Fig. 7. Although the retrieval results of *κ*_a_(*T*) were inaccurate, the calculated temperature response in the right boundary was very close to the results calculated with the real properties. The maximum relative error of the first retrieval was approximately −0.03%, which was slightly higher than that of the second retrieval. The distribution of the temperature in the media was also not sensitive to *κ*_a_(*T*). The temperature distribution calculated by the real and estimated properties matched with each other very well (see Fig. 8). This finding indicated that the optical properties could not be estimated accurately regardless of where or when the thermal information (temperature) was measured. The accuracy of the retrieval results would not be improved by only introducing additional thermal information even when the temperature change inside the media was measured. Therefore, the optical and thermal signals in the inverse problem must be included to retrieve the temperature-dependent absorption coefficient and thermal conductivity accurately.

## Conclusion

In the present work the constant absorption coefficient *κ*_a_ and thermal conductivety *λ* are estimated by using the temperature response in the right side of the 1D slab. The influence of the measurement errors was considered. Then, the temperature-dependent properties were retrieved. However, it was found that *κ*_a_(*T*) could not be estimated accurately through temperature signals only. Therefore, a secondary method using the information of both thermal and optical signals was proposed to estimate *κ*_a_ and *λ*. The retrieval results showed that the temperature-dependent *κ*_a_ and *λ* could be estimated accurately. The following conclusions can be drawn:

1. The temperature-independent absorption coefficient *κ*_a_ and thermal conductivety *λ* can be estimated accurately by SPSO algorithm even with measurement errors.
2. The temperature responses were not sensitive to *κ*_a_, whereas they were highly sensitive to *λ*. The radiative responses were highly sensitive to *κ*_a_ but not very sensitive to *λ*.
3. The optical and thermal signals should be considered synthetically to estimate the temperature-dependent properties of the transparent media.
4. The temperature-dependent *κ*_a_ and *λ* can be estimated accurately by using the proposed secondary optimization method.

Further study will focus on the application of the proposed method to the micro- or nano-scale coupled radiation and conduction problems.

## Additional Information

**How to cite this article**: Ren, Y. *et al.* Simultaneous retrieval of temperature-dependent absorption coefficient and conductivity of participating media. *Sci. Rep.* **6**, 21998; doi: 10.1038/srep21998 (2016).

## Figures and Tables

**Table 1:** Parameter settings of the test case.

Thickness *L* [m]	0.01
Heat capacity *ρc*_p_ [J/(m^3^·K)]	2.2 × 10^6^
Thermal conductivity *λ* [W/(m·K)]	0.7
Convective heat transfer coefficient *h*_w_ [W/(m^2^·K)]	7.0
The initial (ambient) temperature *T*_s_ [K]	300
Pulse width *t*_p_ [s]	1.0
Laser intensity *q*_la_ [W/m^2^]	50,000
Absorbing coefficient *κ*_a_ [m^−1^]	1.0
Scattering coefficient *σ*_a_ [m^−1^]	0.0
Refraction index *n*_1_	1.5

**Table 2:** Estimated results of *κ*_a_ and *λ* with and without measurement errors.

	*κ*_a_ [m^−1^]	*ε*_rel_ [%]	*λ* [W/(m·K)]	*ε*_rel_ [%]
*γ* = 0	18.700	0.000	0.610	0.000
*γ* = 1	18.764	0.340	0.610	0.056
*γ* = 3	18.891	1.021	0.609	0.169
*γ* = 5	19.018	1.703	0.608	0.281

**Table 3:** Retrieval results of the temperature-dependent absorption coefficient and thermal conductivity.

Parameters	*a*_1_ = −22.1	*a*_2_ = 0.23	*b*_1_ = 0.012	*b*_2_ = 4.09 × 10^−4^
Estimated value	−6.638	0.180	0.012	4.090 × 10^−4^
Relative error [%]	69.964	21.661	0.050	0.008

**Table 4:** Second retrieval results of the temperature-dependent absorption coefficient.

Parameters	*a*_1_ = −22.1	*a*_2_ = 0.23
Estimated value	−22.114	0.230
Relative error [%]	0.063	0.017
